# Spatial-genetic structuring in a red-breasted merganser (*Mergus serrator*) colony in the Canadian Maritimes

**DOI:** 10.1002/ece3.10

**Published:** 2011-10

**Authors:** David J Fishman, Shawn R Craik, David Zadworny, Rodger D Titman

**Affiliations:** 1 Department of Wildlife, Fisheries and Aquaculture, Mississippi State UniversityMississippi 39762; 2 Département des Sciences, Université Sainte-AnnePointe-de-l'Église, NS, B0W1M0, Canada; 3 Department of Animal Science, McGill UniversitySte-Anne-de-Bellevue, QC, H9X3V9, Canada; 4 Department of Natural Resource Sciences, McGill UniversitySte-Anne-de-Bellevue, QC, H9X3V9, Canada

**Keywords:** Genetic-spatial autocorrelation, kin-associations, *Mergus serrator*, microsatellites, nesting synchrony, red-breasted merganser

## Abstract

The clustering of kin is widespread across the animal kingdom and two of the primary mechanisms underlying the formation of these patterns in adult kin are (1) philopatric tendencies and (2) actively maintained kin associations. Using polymorphic microsatellites, we had set out to characterize the level of genetic-spatial organization within a colony of female red-breasted mergansers (*Mergus serrator*) breeding on a series of small barrier islands in Kouchibouguac National Park, NB, Canada. Additionally, using nesting data from this colony, we explored possibilities for the existence of kin associations and/or cooperative interactions between these individuals; specifically in the form of the synchronization of breeding activities (i.e., incubation initiation). Our results include: (1) the detection of broad-scale genetic structuring over the entire colony, as females nesting on separate islands were to some extent genetically distinct; (2) the detection of weak, yet significant, positive spatial autocorrelation of kin at the fine scale, but only in the more densely-populated areas of this colony; and (3) the synchrony of breeding activities among proximally nesting females, apart from any factors of relatedness. While these results confirm the existence of genetic-spatial organization within this colony, the underlying mechanisms producing such a signal are inconclusive.

## Introduction

Certain settling decisions result in the formation of clusters of kin in space. Two primary mechanisms underlying the formation of these associations in adult kin are (1) philopatric tendencies and (2) kin associations (Van der Jeugd et al. 2002; Sonsthagen et al. 2010). In the former, kin clustering is merely a biproduct of the common preference of related individuals to settle in proximity to their natal territory (Greenwood 1980). This drive allows individuals to maximize advantages associated with site familiarity (Wright 1943; Rathbun 1979; Beletsky and Orians 1991). Conversely, kin associations are direct efforts by animals to surround themselves with other genetically similar individuals. By doing so, the emergence of within-group cooperative and altruistic behaviors is facilitated (Reyer 1984; McAllister and Roitberg 1987) and opportunities for kin selection are enhanced (O'hara and Blaustein 1981; Nituch et al. 2008). Interactions between kin can result in numerous benefits including increased survivorship (Lambin and Krebs 1993), local recruitment (Stoen et al. 2005), and reproductive success (Andersson and Ahlund 2000; Nielsen et al. 2006). Philopatry and kin associations are by no means mutually exclusive and it is even suggested that natal philopatry is a precursor for the emergence of kin associations (Greenwood 1980).

In recent years, due to the increased accessibility of genetic markers, the spatial structuring of kin, that is, genetic-spatial autocorrelation (GSA), has been shown to be widespread across various animal taxa; from mammals (Girman et al. 1997; Taylor et al. 1997; Coltman et al. 2003), to arthropods (Burgman and Williams 1995; Loeb et al. 2000; Uesugi et al. 2009), to birds (Piertney et al. 1998; Shorey et al. 2000). Given the philopatric nature of female waterfowl (Doty and Lee 1974; Cooke et al. 1975), it is expected that colonially nesting species, for example, common eiders (*Somateria mollissima*) and red-breasted mergansers (*Mergus serrator*), exhibit some degree of GSA (Pearce 2007). Kin associations have also been reported to occur among certain waterfowl (Andersson and Ahlund 2000; Nielsen et al. 2006); and accordingly, such behaviors should also contribute to the presence of GSA (Van der Jeugd et al. 2002; McKinnon et al. 2006; Waldeck et al. 2008; Sonsthagen et al. 2010). Kin associations within a colony are expected to result in genetic structuring at the local scale (Sonsthagen et al. 2010); however fine-scale GSA is not an exclusive indicator of this phenomenon. The presence of (1) high levels of intracolonial relatedness and/or (2) extreme philopatric tendencies, will also produce this signal (Van der Jeugd et al. 2002; Sonsthagen et al. 2010). In the first case, fine-scale GSA can occur randomly if there is a high level of background relatedness (Fowler et al. 2004). For example, this was believed to have occurred among greater white-fronted geese (*Anser albifrons frontalis*) where nests of kin are occasionally found in tight clusters (Fowler et al. 2004). High intracolonial relatedness can result from a high proportion of females with philopatric tendencies (Greenwood 1980; Ratnayeke et al. 2002). Second, the extent of the philopatric tendencies exhibited by colonial females will have consequences on the degree of GSA observed (Van der Jeugd et al. 2002; Sonsthagen et al. 2010). For instance, first-order relatives (i.e., mother–daughter or sister –sister) exhibiting fidelity to a particular site (e.g., a nest bowl) would result in a stronger, more acute signal of GSA than females returning merely to a given region. While the manifestations listed above may not be easily deconfounded, complementing observations of fine-scale GSA with other ecological data, specifically that of brood synchrony, can bring greater meaning to its interpretation by further qualifying the relationships between kin (i.e., not just with respect to geographical distance).

The red-breasted merganser (*M. serrator*) is a medium-size sea duck that breeds across the Holarctic range. Strong philopatric tendencies of breeding females have been reported for colonial populations, including on Tern Islands (TIs) at Kouchibouguac National Park, New Brunswick through mark-and-recapture studies (R. D. Titman, pers. comm., see appendix of Craik 2009). While, it is therefore expected that at least some degree of genetic structuring exists (Pearce 2007), the extent and scale at which this occurs is currently unknown. Furthermore, the potential presence of kin association between nesting females remains untested.

The main objective of our study is to examine patterns of spatial-genetic organization among nesting hens on the TIs at various scales. Using a multivariate approach, we first assessed the degree of genetic structuring with respect to the island females were nesting on (i.e., broad scale). Second, we examined the correlation between the geographic and genetic distances between nesting females (i.e., local scale). Finally, in order to assist with our interpretation of GSA, we measured the timing of a female's nesting activities and related these observations to both the geographic and genetic distances to nearby females. To our knowledge, this is the first study to (1) look at the levels of genetic structuring with a red-breasted merganser colony and (2) examine GSA directly in conjunction with the synchronization of nesting activities.

## Methods

### Field collection

**Figure 1:**
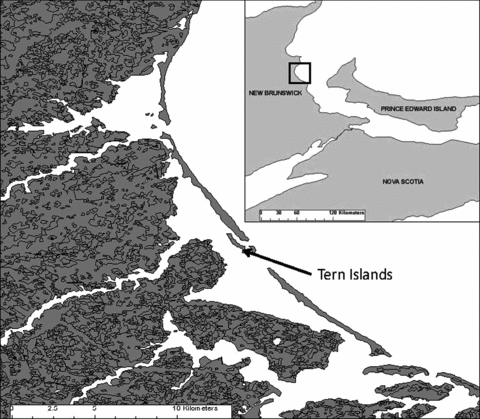
The study population of nesting red-breasted mergansers was located on a series of small barrier islands, collectively known as the Tern Islands (TIs), situated in Kouchibouguac National Park, New Brunswick, Canada. Field data collection was carried out in the summer months of 2008. The ESRI shapefiles were obtained from GeoBase (http://www.geobase.ca/).

The TIs consists of two small barrier islands: Tern Island A (TI-A) and Tern Island B (TI-B) (Fig. 1). Field data were collected during June and July 2008. Systematic searches were conducted on a weekly basis throughout the incubation period to locate as many nests as possible. Red-breasted merganser nests are lined with vegetation and plucked feathers and are situated primarily in marram grass (*Ammophila breviligulata*) (Bent 1962; Craik and Titman 2009). The coordinates of each nest were recorded using a global positioning system (GPS model eTrex, Garmin Ltd., Olathe, KS, USA). The incubation stage (d) of each nest was assessed by floating eggs as described by Westerskov (1950) and the final clutch sizes of each nest were recorded. Efforts to trap females commenced only in the final quarter of the incubation period to minimize risk of nest abandonment. Females were captured using nest traps (Weller 1957) and from each captured bird, a 0.25 cc blood sample was collected from the ulnar vein and deposited in a heparinized Vacutainer® (BD, Franklin Lakes, NJ, USA). Each Vacutainer was labeled accordingly and eventually stored at –20°C. Once a nest was deemed inactive (i.e., either due to success in hatching, predation, or abandonment), four to eight contour feathers were recovered from the bowl, placed in a small envelope, and stored at 4°C.

### Lab protocol

DNA extraction protocols for both blood and feather samples were initiated approximately 6 months after they had been collected. The extraction and precipitation of DNA from blood samples was conducted using a DNeasy® Blood and Tissue Kit (QIAGEN) and from each sample 10 µl of blood was used. Procedures were carried out in accordance with the manufacturer's recommendations with the exception to having used (1) a PBS solution with pH = 7.4 as opposed to pH = 7.2 and (2) an extended incubation period of 30 min after step 2 of the protocol. The protocol for extraction and precipitation of DNA from feathers was based on the phenol–chloroform method described by Sambrook et al. (1989).

Eight primer pairs were used in the polymerase chain reactions (PCR): *Aph08, Aph13*, *Aph15*, *Aph20*, *Aph24*, *Mm01*, *Mm04*, and *Hhiµ5*; the forward primer of each pair was tagged with one of the following fluorescent labels: 6FAM, VIC, NED, or PET (Applied Biosystems). PCRs were carried out using a PTC-200 thermocycler (MJ research Inc.). Each reaction had 100 ng of DNA, 0.25 µM each of forward and reverse primer, 2 µM of MgCl_2_, and a total reaction volume of 25 µl. All primers were processed under the same thermal-cycling regime: initialization (94°C for 2 min), 40 cycles of denaturation (94°C for 15 s), annealing (50°C for 15 s), and elongation (72°C for 30 s); and an extended elongation period (72°C for 30 min). The products obtained from the PCR were subsequently resolved using an ABI-3730XL DNA Analyzer (Applied Biosystems).

### Analysis of genetic diversity

Allelic frequencies, Hardy–Weinberg equilibria (HWE) (using Markov chain method with default parameters), and linkage disequilibrium were calculated using Genepop 3.1 (Raymond and Rousset 1995). Because no microsatellite primers developed specifically for the *M. serrator* genome are currently available, our genetic analysis was limited to the use of heterologous primers. Deviations in HWE, specifically an excess of homozygosity, can be indicative of the presence of nonamplifying alleles (Pemberton et al. 1995). Nonamplifying alleles can be prevalent especially when using heterologous primers, and if there is a paucity of detectable allelic variability, their presence may obscure the analysis (Pemberton et al. 1995). Therefore, in our study, an HWE-deviated locus was required to have at least three detectable alleles before it was integrated into our approximation of genetic distances.

A rarefaction analysis, using the Queller and Goodnight (1989) coefficient of relatedness, was employed to assess the consistency of the estimates of relatedness. Queller & Goodnight's coefficient of relatedness (*R*) is a pairwise estimate of kinship obtained by first weighting alleles by their respective frequencies so that the rarer an allele, the greater its weight. The coefficient of relatedness of individual *x* to individual *y* can be defined as:

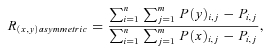
(1)where *n* is the total number of loci, *m* is total number of allelic positions (i.e., 2 for diploid organisms), *P*_*i*,*j*_ is the population frequency of the allele at the *i*th locus and the *j*th allelic position in individual *x*, *P*(*x*)_*i*,*j*_ is the frequency of that allele within individual *x* (i.e., either 0.5 for heterozygotes or 1 for homozygotes), and *P*(*y*)_*i*,*j*_ is the frequency of that allele in individual *y* (i.e., either 0, 0.5, or 1). This index is asymmetrical as *R*_(*x*,*y*)_≠*R*_(*y*,*x*)_ and therefore in order to obtain symmetrical pairwise coefficients, the numerator and denominator values from Equation (1) for *R*_(*x*,*y*)_and *R*_(*y*,*x*)_were summed prior to division. The rarefaction algorithm consisted of the following steps: (1) randomly sample (without replacement) a set of microsatellites; (2) calculate relatedness based solely on that marker; (3) randomly sample an additional marker; (4) recalculate relatedness using both markers; (5) calculate the absolute value of the difference between the estimates produced in step 2 and 4; and finally (6) repeat steps 1–5 until all markers have been sampled. This algorithm was repeated 1000 times and the means differences between each estimate were calculated. This procedure was carried out using the web-based analysis tool, RE-RAT (http://people.musc.edu/~schwaclh/).

### Analysis of GSA

Nest densities on each island were calculated by dividing the number of nests on an island by its total area (m^2^). Our measurement of area was based on the island's perimeter and was computed using ArcGIS (ESRI Inc.). Euclidean distances were calculated between nests using geographic coordinates (UTM). The Euclidean distance between two points is defined by
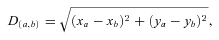
(2)where (*x_a_*, *y_a_*)are the *x*- and *y*-coordinates of nest *a*, and (*x_b_*, *y_b_*)are the *x*- and *y*-coordinates of nest *b*, respectively. The multivariate approach described by Smouse and Peakall (1999) was employed as a measure of genetic distance between nesting females. At a given microsatellite locus with four alleles (*A*, *B*, *C*, *D*), the distance between two individuals is defined as: *d*(*AA*, *AA*) = 0; *d*(*AB*, *AB*) = 0; *d*(*AA*, *AB*)= 1; *d*(*AB*, *AC*) = 1; *d*(*AB*, *CD*) = 2; *d*(*AA*, *BC*) = 3; *d*(*AA*, *BC*) = 4. Pairwise distances were first calculated for each locus separately and then summed across all loci. Factors that influenced our selection of this coefficient included its preestablished compatibility with multivariate statistics as well as its precedence in similar studies that explored GSA among colonial waterfowl (e.g., Sonsthagen et al. 2010). One risk associated with the use of this coefficient is that errors, arising from the presence of nonamplifying alleles, are inflated because the distances between homozygous pairs are scored as greatest. We feel that this risk has been minimized given our criteria for reducing the likelihood of nonamplifying alleles in our dataset (see above) and that the advantages of using this particular coefficient outweigh such risks. The above genetic distances were assembled into a matrix and integrated into a principal coordinate analysis (PCoA) in order to assess patterns of genetic variation across the islands. An ordination diagram was produced by plotting the first two eigenvectors against each other. Additionally, a single-linkage cluster analysis of the first two principal coordinates was carried out and superimposed onto the ordination diagram. By doing so, the distances between objects were further resolved.

The detection of GSA on each island was achieved using a Mantel correlogram (Legendre and Legendre 1998). The Mantel correlation coefficient (*r*) behaves similar to other correlation coefficients and ranges from –1 to 1. Positive coefficients indicate the presence of clustering, while negative coefficients indicate dispersion between objects. This technique partitions geographic and genetic distance matrices into numerous subdistance classes (based on only one of the distance matrices) and calculates the Mantel correlation coefficient (*r*) for each pair of corresponding submatrices, thereby facilitating the detection of nonlinear trends in the data (Burgman and Williams 1995; Skabo et al. 1998). The distance classes, by which these submatrices are defined, constitute an important user-defined aspect of this analysis; depending on the classes selected, different patterns of correlation may be observed. In an effort to facilitate additional comparisons, we selected distance classes of 10-m intervals. In order to remove dependence on normality, all tests were based on the Spearman rank statistic. The significance of *r* was determined by comparing the reference value to a distribution of 9999 values, which were generated from random permutations of the data. The null hypothesis tested was that the associations described in the real dataset are just as likely to be found in randomly generated data. Holm's (1979) correction for multiple testing was applied and a critical value of α= 0.05 was used.

### Nesting synchrony

Dates were converted into Julian days; 1 May was arbitrarily designated as day 1. Incubation-initiation dates for each nest were calculated by subtracting the recorded incubation stage from the date on which it had been assessed. Nest-initiation dates were estimated by subtracting the reported laying interval for red-breasted mergansers (1.5 d) times the total number eggs from the estimated incubation-initiation date (Titman 1999). However estimates of nest initiation were only back-dated to a maximum of 18 d (1.5 × 12 eggs) assuming that nests with ≥13 eggs had been parasitized as per the criterion used by Craik and Titman (2009).

Mean dates of nest initiation and incubation initiation for the entire colony were computed and compared between each island using the Kruskal–Wallis rank-sum test (Siegel and Castellan 1988). The null hypotheses were that the mean nest-initiation date and incubation-initiation date were equal across islands. Significance of the test statistic was assessed using a critical value of α= 0.05. The degree of shared explanatory power between the two covariates was assessed by first regressing one against the other and then calculating the coefficient of determination (*R^2^*). Either of these covariates can be justified as a proxy for nesting synchrony. However, given the objectives of this study, we carried out our analyses using incubation initiation as it seemed to be a more fitting measurement of the overlap of nesting activities.

Mantel correlograms were used to assess the degree of correlation between (1) incubation initiation and genetic distance (Smouse and Peakall 1999) and (2) incubation initiation and geographic distance. The former was used to further qualify the relationship (i.e., with respect to the synchronization of nesting activities) between the nesting females whose genetic distances were known, while the latter provided an indication of the degree of nesting synchrony occurring throughout the entire colony. Because the use of Mantel correlograms requires comparison of two distance matrices, the Euclidean distances (Equation [2]) between nesting females, on the basis of their incubation-initiation dates, were computed also using Equation (2). Distance matrices were partitioned into submatrices of 3-d and 10-m intervals for tests (A) and (B), respectively. The tests were based on the Spearman rank correlation coefficient and the significance of the test statistic was assessed using 9999 random permutations, Holm's corrections, and a critical value of α= 0.05.

Unless otherwise specified, all calculations were carried out using the open-source statistical software R ver. 2.10.1 (http://www.r-project.org/); Mantel tests and correlograms were computed using the package VEGAN ver. 1.17-2.

## Results

### Field and lab protocol

**Figure 2:**
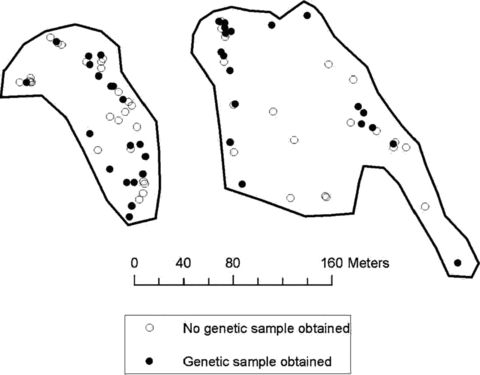
A schematic representation of the red-breasted mergansers nesting on the TIs. The nesting density of TI A (left-hand side) and TIs B (right-hand side) was 0.005 and 0.002 nest/m^2^, respectively. Of the 88 nests discovered, a total of 39 (44.3%) were successfully genotyped (represented by solid circles).

Altogether, 88 nests were located across the TIs. The incubation status was calculated for 75 nests and genetic material was obtained from 60 nesting females. Considering the extensive nature of the search effort, it is very likely that most nests were found. Of 42 DNA samples originating from blood, 33 (79%) were successfully genotyped. In contrast, only six (33%) of 18 DNA samples from feathers were amplified successfully. Therefore, a total of 39 individuals, corresponding to approximately 44% of the total number of nests detected, were genotyped across all eight microsatellite loci. A schematic diagram of the spatial distribution of discovered and genetically sampled nests is presented in Fig. 2.

### Analysis of genetic diversity

The total number of alleles, observed heterozygosity (*H*_O_), expected heterozygosity (*H*_E_), as well as the probability that the null hypothesis (i.e., there no deficiency of heterozygotes) is true for each locus is presented in Table 1. All loci except for *Aph08* and *Aph24* were found to have heterozygote deficiencies. As the number of alleles found at loci *Aph15* and *Hhiµ5* were below the chosen threshold of 3 (see above), they were excluded from subsequent analyses. None of the loci were found to be in linkage disequilibrium.

**Table 1:** The total number of alleles, observed heterozygosity (*H*_O_), and expected heterozygosity (*H*_E_) for each microsatellite locus. All loci except for *Aph08* and *Aph24* have heterozygote deficiencies (in italics). Loci *Aph15* and *Hhiµ5* were excluded from subsequent analyses due to their lack of allelic variability

Primer name	Number of alleles	*H*_O_	*H*_E_	Prob. (*H1*= heterozygote deficiency)	GenBank accession number	Publication
Aph08	5	0.62	0.63	0.506	AJ515887	Maak et al. 2003
*Aph13*	*6*	*0.64*	*0.77*	*0.009*	*AJ515889*	–
*Aph15*	*2*	*0.00*	*0.44*	*0.000*	*AJ515890*	–
*Aph20*	*4*	*0.46*	*0.48*	*0.001*	*AJ515895*	–
Aph24	6	0.44	0.50	0.071	AJ515899	–
*Mm01*	*5*	*0.18*	*0.23*	*0.017*	*AY679118*	Gautschi and Koller 2005
*Mm04*	*13*	*0.36*	*0.82*	*0.000*	*AY679121*	–
*Hhiµ5*	*2*	*0.00*	*0.05*	*0.014*	*AF025903*	Buchholz et al. 1998

**Figure 3:**
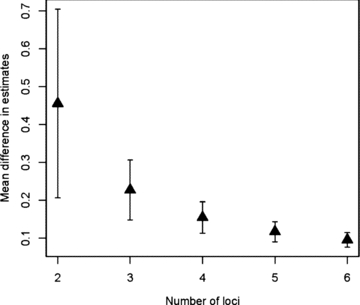
A rarefaction analysis depicting the relationship between the number of loci used and the resultant Queller and Goodnight (1989) estimate of relatedness. Each point represents the mean difference between current estimate of relatedness and the one previous to it; the bars represent the standard deviation. A total of 1000 permutations were used to generate these data.

The Queller & Goodnight coefficients of relatedness were calculated for each pair of nesting females within the colony, and the mean estimate of relatedness was –0.026 ± 0.281 (SD). Rarefaction analysis showed that with each additional locus, both the mean difference and variance between estimates of relatedness decreased dramatically (Fig. 3). The addition of more loci would further reduce inconsistencies in the estimated coefficients, albeit by miniscule amounts. Therefore, while our estimates of relatedness and genetic distance are not precise, obtaining a proxy of relatedness was of greater importance to the objectives of this study. Thus, we consider that the level of precision obtained is satisfactory.

### Analysis of GSA

**Figure 4:**
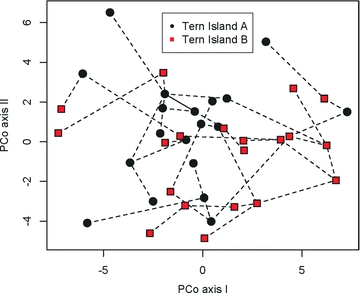
Two-dimensional PCoA ordination diagram. The proportion of variance explained by the first two principal components is 0.265 and 0.139, respectively. In this figure, the positions of the objects (i.e., nesting females) in relation to one another are approximations of their genetic distances. The measure of genetic distance used in the PCoA was that described by Smouse and Peakall (1999). The results from single-linkage cluster analysis are superimposed onto this ordination plot (represented by dotted lines) as an additional means for assessing the associations between objects.

The density on TI-A and TI-B was 0.05 and 0.02 nests/m^2^, respectively. The cumulative proportion of variance in the genetic data accounted for by the first three principal coordinates is 0.539. Patterns of genetic structuring in relation to a female's island of origin were identified (Fig. 4). Proximities between points (i.e., nesting females) in this diagram are approximations of their genetic distances; the closer together they are in ordination space, the more genetically similar they are.

**Figure 5:**
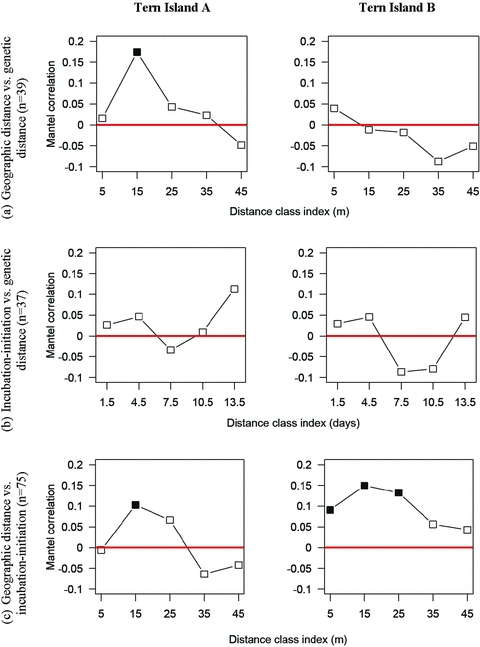
The Mantel correlograms for TI-A and TI-B. Significant distance intervals are emphasized with a solid point in the diagram. A null correlation is represented by a solid red line in each panel. The corresponding figures to these panels are presented in Table 2. Significant values were determined by generating 9999 random permutations of the data and all *P*-values were corrected for multiple testing using Holm's method. A critical value of α= 0.05 was applied.

Weak, yet significant, GSA was detected on TI-A (Table 2A). Specifically, females nesting within 10–20 m from one another on that island were found to be more related to each other than expected by chance (*r*= 0.174, *N*= 32, corrected *P*-value = 0.028). No significant correlations, at any of the distance intervals tested, were found on TI-B (corresponding correlograms are given in Fig. 5A).

**Table 2:** The distance intervals, number of pairwise observations within a given interval, Mantel correlation coefficients (*r*), and associated *P*-values for the distances being compared. The probabilities were generated using 9999 random permutations of the data and all *P*-values were corrected for multiple testing using Holm's method. A critical value of α= 0.05 was required before rejecting the null hypothesis. Only the first three distance classes of each test are shown here as there were no significant or marginally significant values detected beyond this. The units of measurement for both (A) and (C) are meters while that for (B) is days

	Distance interval (DI)	No. observations	Mantel correlation (*r*)	*P*-value (corr.)
(A) Geographic distance versus genetic distance (*n*= 39); DI in (m)				
TI-A	[0–10[	12	0.016	0.405
	[10–20[	32	0.174	0.028*
	[20–30[	34	0.043	0.534
TI-B	[0–10[	34	0.040	0.333
	[10–20[	18	−0.011	0.665
	[20–30[	24	−0.018	0.998
(B) Incubation-initiation date versus genetic distance (*n*= 37); DI in (d)				
TI-A	[0–3[	72	0.026	0.367
	[3–6[	46	0.046	0.653
	[6–9[	82	−0.034	0.980
TI-B	[0–10[	122	0.030	0.482
	[10–20[	76	0.046	0.064
	[20–30[	58	−0.087	0.661
(C) Geographic distance versus incubation-initiation date (*n*= 75); DI in (m)				
TI-A	[0–10[	82	−0.006	0.455
	[10–20[	96	0.103	0.022*
	[20–30[	100	0.067	0.139
TI-B	[0–10[	78	0.091	0.049*
	[10–20[	36	0.150	0.000***
	[20–30[	54	0.132	0.001***

*** 0.001

** 0.01

* 0.05

### Nest-initiation and incubation synchrony

Over the entire colony, the mean nest-initiation date was 7 June (range = 15 May–15 July) and, on average, incubation began approximately 13 d later; no significant differences in either of these variables were found across the islands (Table 3). The proportion of variance (*R^2^*) for “nest-initiation” date accounted for by “incubation-initiation” date was 0.896 implying that there is a high proportion of shared information between them (*b*= 1.317, *P*-value ≤ 0.000).

**Table 3:** Mean dates of nest initiation and incubation initiation for red-breasted mergansers across the TIs. The days were transformed into Julian days using 1 May as day 1. In both tests, the null hypothesis, stating that the variable did not differ between islands, could not be rejected. A critical value of α= 0.05 was used

Variable name	Mean of TI-A	Mean of TI-B	Kruskal–Wallis (χ^2^)	df	*P*-value
Nest-initiation date	36.25	38.78	0.719	1	0.396
Incubation-initiation date	49.63	51.23	0.405	1	0.524

There was no spatial structuring detected between genetic-distance and incubation-initiation date across any of the intervals tested on either of the two islands (Table 2B; Fig. 5B) (*n*= 37; two samples lost because of missing incubation data).

Unlike the comparison with genetic distance, significant positive Mantel correlations were detected between geographic distance and incubation-initiation date across the entire colony (*n*= 75) (Table 2C; Fig. 5C). On TI-A, positive correlations were observed between females nesting within 10–20 m from one another (Mantel *r*= 0.103, *N*= 96, *P*-value = 0.022). This trend was more extensive on TI-B where synchrony was detected between those within a 0–30 m distance interval (i.e., consisting of three contiguous intervals) (Table 2C).

## Discussion

### Genetic-spatial autocorrelation

Our study revealed some degree of genetic structuring among females in relation to where they nested, despite the close proximity of the two islands (Fig. 4). Positive fine-scale spatial autocorrelation of kin was revealed with this colony, albeit at low frequencies and only on the more densely populated TI-A (Fig. 5A). Such trends on TI-B were absent.

The colony-scale structuring observed suggests that certain females, along with their brood mates, prefer to nest on their islands of origin. Such inferences are further supported by numerous field observations (R. D. Titman, unpubl. data). Exhibiting fidelity to one's natal island may be a rule-based mechanism to maximize the likelihood of choosing good-quality nesting and brood-rearing habitat (Schjorring 2001). In several species, juvenile experience can serve as a basis for future settling decisions (Brown and Brown 1992; Osorio–beristain and Drummond 1993; Schjorring et al. 2000).

The strength of fine-scale GSA is expected to be weak (Sonsthagen et al. 2010). Interference with the signal of GSA can be the result of ecological and/or sampling-related factors. Ecological factors include: (1) constraints on nest-site selection and (2) mechanism of recognition. In the first case, constraints on nest-site selection can diminish the presence of GSA, since where an animal nests has important fitness consequences resulting from access to resources and varying degrees of exposure to predation, brood parasitism, and the elements (Clark and Shutler 1999). It is known that vegetation type and structure, in fact, have a strong influence on nest-site selection in this colony of mergansers (Craik and Titman 2009). The second factor is only applicable where GSA is attributed to the presence of kin associations. Kin associations require that the individuals involved are capable of recognizing each other (Waldman 1988; Andersson and Ahlund 2000). If mechanisms of kin recognition are based on familiarity as opposed to phenotype matching (e.g., Petrie et al. 1999; Van der Jeugd et al. 2002), females may actually be attempting to nest in proximity to brood mates and not siblings, *per se*. Therefore, the presence of high rates of intraspecific brood parasitism within a colony may disrupt the signal of GSA, since not all of one's neighbors are necessarily first-order kin. This consideration is noteworthy because brood parasitism within the TIs can reach up to 40% in some years (Young and Titman 1988). Alternatively, sampling-related interference may have resulted from (1) our underlying assumption that the females (or feathers) apprehended from a given nest are endemic to it and (2) a paucity of genetic samples obtained from nests. While it cannot be discounted as a possibility that the signal on TI-B was lacking due to the sampling efforts (i.e., only approximately 40% of nests were sampled across the colony), it is noteworthy that there are not even vague patterns of positive correlation observed within the correlogram for that island (Fig. 5A). In any case, as the ratio of sampled verse unsampled nests on both islands was virtually the same, it could be reasoned that the degree of structuring on TI-B, if any at all, is much less than on TI-A. One possible explanation for why no GSA was detected in the 0–10 m distance interval might relate to our finding that no nesting synchrony exists between kin (see discussion below). If this is the case, proximally nesting kin most probably colonized a given area in multiple waves. As a function of time, the vacancy of suitable nest sites will be reduced due to the nesting efforts of non-kin and thereby posing a heightened constraint to how close together kin are able to settle.

As stated in the introduction, the detection of fine-scale GSA is ecologically inconclusive. Extreme philopatry, high intracolonial relatedness, and kin associations are three possible phenomena, which the clustering of kin at the local scale can be attributed to. Sonsthagen et al. (2010) were able to disqualify the possibility that extreme philopatry was the mechanism underlying the local-scale clustering of kin in colonies of Pacific common eiders (*S. m. v-nigrum*). They reasoned that because of the seasonal instability of nests caused by perpetual movements of driftwood, the likelihood that females exhibit fidelity to a particular bowl is minimal. Instead, the patterns of association they observed could more likely be attributed to the presence of kin associations (Sonsthagen et al. 2010). While it was observed in our study that at least some females exhibited fidelity to their natal island, the results are contrary to what is expected if every female had the proclivity to return to a specific natal site (i.e., *extreme philopatry*), namely that fine-scale GSA was only detected on TI-A. Therefore, the patterns of GSA observed within the TIs are unlikely to be solely the result of extreme philopatry. Furthermore, due to the permutation-based statistical methods employed in our study, the assessments made had effectively accounted for the likelihood of observing a given pattern of the spatial distribution of nests. Consequently, the possibility that the pattern observed is only an artifact of high levels of intracolonial relatedness can be largely discounted.

The third tentative factor that may contribute to the detection of GSA is the presence of kin associations. Feasible forms of kin association within this colony include: (1) lowered aggressive tendencies toward neighboring kin; (2) prehatch brood amalgamation (pre-HBA); (3) posthatch brood amalgamation (post-HBA); and (4) cooperative defense. Reduced agonism between kin can be important in the recruitment of related individuals into a given area (MacColl et al. 2000; Hoglund and Shorey 2003), especially under high-density conditions where the frequencies of interaction between individuals are high (Sonsthagen et al. 2010). Generally speaking, it has been demonstrated that the greater the proximity between neighbors, the greater their vulnerability to thievery (Wojcieszek et al. 2007), cannibalism (Brown 1967; Yom–Tov 1974 and references therein), and brood parasitism (Reyer 1984; McRae 1998); presumably as a function of increased nest access. Most relevant to the TIs is likely to be intraspecific brood parasitism (i.e., pre-HBA), which is incidentally known to have occurred at some of the highest rates known among ground-nesting waterfowl (Young and Titman 1988). Aggression toward foreign females would be expected if brood parasitism has a negative effect on host fitness (e.g., Milonoff et al. 2004) and under such circumstances, both recognition and discriminatory behavior toward kin would serve as adaptive traits (Andersson 2001; Lopez–Sepulcre and Kokko 2002). Under such a scenario, by helping kin to obtain a nearby nesting territory, the costs associated with parasitism are offset by inclusive fitness obtained from either (1) the offspring that are reared directly by the recruited individual or (2) as a result of increased level of relatedness to parasitic eggs. While a higher level of relatedness between donors and recipients has been described in some populations of goldeneyes (*Bucephala clangula*) (Andersson and Ahlund 2000) and common eiders (Andersson and Waldeck 2007; Waldeck et al. 2008), the relationship between the two in this colony of red-breasted mergansers has yet to be described. The prospect that females on the TIs exhibit increased tolerance toward related females is intriguing, although with no direct line of support. However, the observation that positive GSA was prominent only on TI-A, which also had the higher nesting density, is consistent with the hypothesis that there is an increased tolerance by females toward kin who are attempting to establish adjacent nests. Conversely, the prospect of kin-based post-HBA in this colony has not been well explored. Aside from a few accounts (e.g., Bukacinski et al. 2000; Kraaijeveld 2005), the evidence that a higher degree of relatedness exists between nonparental females and the young they are tending, has not been well substantiated. Instead, more evidence seems to implicate accidental fusion (Gorman and Milne 1972; Savard 1987) and abandonment (Pöysä 1995; Öst 1999; Kilpi et al. 2001) as the two principal factors driving adoption and post-HBA. Öst et al. (2005) showed explicitly that female relatedness was not a factor influencing patterns of brood amalgamation among eider hens. This assertion cannot be made here, as the possibility of relatedness being a factor is unclear. While on one hand, there was a lack of incubation synchrony observed between kin (Table 2C), the brood-rearing period of red-breasted mergansers is known to be long (≈7 weeks) and amalgamated broods of mixed cohorts have been observed (S. R. Craik, pers. obs.). Finally, the possibilities of kin-based cooperative defense operating within this colony are discussed below.

### Nesting synchrony

In this study, we report synchrony of incubation between close-nesting females throughout the entire colony (Table 2C), albeit not necessarily amongst kin (Table 2B). While these observations are consistent with the hypothesis presented above that females are engaging in a cooperative-defense strategy (i.e., through deliberate synchronization of nesting activity), it cannot be ruled out that the synchrony observed is in fact just a biproduct of the gradual thawing of suitable habitat.

Nesting in groups has several defensive advantages including benefits from (1) the vigilance and predator-detection abilities of group mates; (2) the defensive actions taken against predators by group mates; and (3) predator satiation from the offspring of group mates (Schmutz et al. 1983; McKinnon et al. 2006). Synchronizing breeding with neighboring females essentially maximizes the period of nesting overlap and, therefore, simultaneously maximizes the period of time when such advantages are obtained (Schmutz et al. 1983). Even though the payoff from this type of cooperative behavior is augmented if directed toward kin (O'hara and Blaustein 1981) (and is for all forms of cooperation), if females are surrounded by a significant proportion of nonrelated neighbors, it may then be best to pursue this strategy apart from considerations of genetic relatedness. Furthermore, constraints such as obtaining a sufficient accumulation of endogenous reserves may be more critical to a female's reproductive success as she is deciding when to initiate nesting (Devries et al. 2008).

## Conclusion

Our findings support the hypothesis that spatial-genetic organization exists among the red-breasted merganser hens in this colony. Based on this analysis, it appears that organization is present both at the regional (i.e., across islands) and fine scale (i.e., between neighboring nests); however the latter was only detected in the more densely populated regions of the colony. Currently, no concrete inferences about the mechanisms driving these patterns can be made. While our results are compatible with the hypothesis that at least some of the fine-scale GSA observed within this colony is a product of an increased level of tolerance among kin, this needs to be investigated in further depth using data from additional years and other colonies. Similarly, while we have found that proximally nesting females exhibit synchronized incubation periods over the entire colony, it cannot be stated for certain that this is deliberately implemented as a part of a cooperative defensive strategy. Although the inferences that can be made based on our results are limited, a solid framework for future research addressing spatial-genetic structuring, both within and outside of this system, has been established.

## References

[b1] Andersson M (2001). Relatedness and the evolution of conspecific brood parasitism. Am. Nat..

[b2] Andersson M, Ahlund M (2000). Host-parasite relatedness shown by protein fingerprinting in a brood parasitic bird. Proc. Natl. Acad. Sci. U. S. A..

[b3] Andersson M, Waldeck P (2007). Host-parasite kinship in a female-philopatric bird population: evidence from relatedness trend analysis. Mol. Ecol..

[b4] Beletsky LD, Orians GH (1991). Effects of breeding experience and familiarity on site fidelity in female red-winged blackbirds. Ecology.

[b5] Bent AC (1962). Life Histories of North American Wild Fowl.

[b6] Brown CR, Brown MB (1992). Ectoparasitism as a cause of natal dispersal in cliff swallows. Ecology.

[b7] Brown RGB (1967). Breeding success and population growth in a colony of herring and lesser black-backed gulls *Larus argentatus* and *L fuscus*. Ibis.

[b8] Buchholz WG, Pearce JM, Pierson BJ, Scribner KT (1998). Dinucleotide repeat polymorphisms in waterfowl (family Anatidae): characterization of a sex-linked (Z-specific) and 14 autosomal loci. Anim. Genet..

[b9] Bukacinski D, Bukacinska M, Lubjuhn T (2000). Adoption of chicks and the level of relatedness in common gull, Larus canus, colonies: DNA fingerprinting analyses. Anim. Behav..

[b10] Burgman MA, Williams MR (1995). Analysis of the spatial pattern of arthropod fauna of jarrah (*Eucalyptus marginata*) foliage using a Mantel correlogram. Aust. J. Ecol..

[b11] Clark RG, Shutler D (1999). Avian habitat selection: pattern from process in nest-site use by ducks?. Ecology.

[b12] Coltman DW, Pilkington JG, Pemberton JM (2003). Fine-scale genetic structure in a free-living ungulate population. Mol. Ecol..

[b13] Cooke F, MacInnes CD, Prevett JP (1975). Gene flow between breeding populations of Lesser Snow Geese. Auk.

[b14] Craik SR (2009). Habitat use by breeding and molting red-breasted mergansers in the Gulf of St. Lawrence.

[b15] Craik SR, Titman RD (2009). Nesting ecology of red-breasted mergansers in a common tern colony in Eastern New Brunswick. Waterbirds.

[b16] Devries JH, Brook RW, Howerter DW, Anderson MG (2008). Effects of spring body condition and age on reproduction in Mallards (*Anas platyrhynchos*). Auk.

[b17] Doty HA, Lee FB (1974). Homing to nest baskets by wild female mallards. J. Wildl. Manage..

[b18] Fowler AC, Eadie JM, Ely CR (2004). Relatedness and nesting dispersion within breeding populations of Greater White-fronted Geese. Condor.

[b19] Gautschi B, Koller B (2005). Polymorphic microsatellite markers for the goosander (*Mergus merganser*). Mol. Ecol. Notes.

[b20] Girman DJ, Mills MGL, Geffen E, Wayne RK (1997). A molecular genetic analysis of social structure, dispersal, and interpack relationships of the African wild dog (*Lycaon pictus*). Behav. Ecol. Sociobiol..

[b21] Gorman ML, Milne H (1972). Creche behaviour in the common eider *Somateria m. mollissima* L. Ornis Scand..

[b22] Greenwood PJ (1980). Mating systems, philopatry and dispersal in birds and mammals. Anim. Behav..

[b23] Hoglund J, Shorey L (2003). Local genetic structure in a white-bearded manakin population. Mol. Ecol..

[b24] Holm S (1979). A simple sequentially rejective multiple test procedure. Scand. J. Stat..

[b25] Kilpi M, Öst M, Lindström K, Rita H (2001). Female characteristics and parental care mode in the creching system of eiders, *Somateria mollissima*. Anim. Behav..

[b26] Kraaijeveld K (2005). Black swans *Cygnus atratus* adopt related cygnets. Ardea.

[b27] Lambin X, Krebs CJ (1993). Influence of female relatedness on the demography of Townsends vole populations in spring. J. Anim. Ecol..

[b28] Legendre P, Legendre L (1998). Numerical Ecology.

[b29] Loeb MLG, Diener LM, Pfennig DW (2000). Egg-dumping lace bugs preferentially oviposit with kin. Anim. Behav..

[b30] Lopez-Sepulcre A, Kokko H (2002). The role of kin recognition in the evolution of conspecific brood parasitism. Anim. Behav..

[b31] Maak S, Wimmers K, Weigend S, Neumann K (2003). Isolation and characterization of 18 microsatellites in the Peking duck (Anas platyrhynchos) and their application in other waterfowl species. Mol. Ecol. Notes.

[b32] MacColl ADC, Piertney SB, Moss R, Lambin X (2000). Spatial arrangement of kin affects recruitment success in young male red grouse. Oikos.

[b33] McAllister MK, Roitberg BD (1987). Adaptive suicidal-behavior in pea aphids. Nature.

[b34] McKinnon L, Gilchrist HG, Scribner KT (2006). Genetic evidence for kin-based female social structure in common eiders (*Somateria mollissima*). Behav. Ecol..

[b35] McRae SB (1998). Relative reproductive success of female moorhens using conditional strategies of brood parasitism and parental care. Behav. Ecol..

[b36] Milonoff M, Pöysä H, Runko P, Ruusila V (2004). Brood rearing costs affect future reproduction in the precocial common goldeneye *Bucephala clangula*. J. Avian Biol..

[b37] Nielsen CR, Semel B, Sherman PW, Westneat DF, Parker PG (2006). Host-parasite relatedness in wood ducks: patterns of kinship and parasite success. Behav. Ecol..

[b38] Nituch LA, Schaefer JA, Maxwell CD (2008). Fine-scale spatial organization reflects genetic structure in sheep. Ethology.

[b39] O'hara RK, Blaustein AR (1981). An investigation of sibling recognition in *Rana cascadae* Tadpoles. Anim. Behav..

[b40] Osorio-beristain M, Drummond H (1993). Natal dispersal and deferred breeding in the blue-footed booby. Auk.

[b41] Öst M (1999). Within-season and between-year variation in the structure of common eider broods. Condor.

[b42] Öst M, Vitikainen E, Waldeck P, Sundström L, Lindström K, Hollmén T, Franson JC, Kilpi M (2005). Eider females form non-kin brood-rearing coalitions. Mol. Ecol..

[b43] Pearce JM (2007). Philopatry: a return to origins. Auk.

[b44] Pemberton JM, Slate J, Bancroft DR, Barrett JA (1995). Nonamplifying alleles at microsatellite loci—a caution for parentage and population studies. Mol. Ecol..

[b45] Petrie M, Krupa A, Burke T (1999). Peacocks lek with relatives even in the absence of social and environmental cues. Nature.

[b46] Piertney SB, MacColl ADC, Bacon PJ, Dallas JF (1998). Local genetic structure in red grouse (*Lagopus lagopus scoticus*): evidence from microsatellite DNA markers. Mol. Ecol..

[b47] Pöysä H (1995). Factors affecting abandonment and adoption of young in common eiders and other waterfowl—a comment. Can. J. Zool.-Rev. Can. Zool..

[b48] Queller DC, Goodnight KF (1989). Estimating relatedness using genetic-markers. Evolution.

[b49] Rathbun GB (1979). The social structure and ecology of elephant-shrews..

[b50] Ratnayeke S, Tuskan GA, Pelton MR (2002). Genetic relatedness and female spatial organization in a solitary carnivore, the raccoon. Procyon lotor. Mol. Ecol..

[b51] Raymond M, Rousset F (1995). Genepop (Version-1.2)—population-genetics software for exact tests and ecumenicism. J. Hered..

[b52] Reyer HU (1984). Investment and relatedness—a cost-benefit-analysis of breeding and helping in the pied kingfisher (*Ceryle rudis*). Anim. Behav..

[b53] Sambrook J, Fritsch EF, Maniatis T (1989). Molecular Cloning: a laboratory manual.

[b54] Savard JPL (1987). Causes and functions of brood amalgamation in Barrows goldeneye and bufflehead. Can. J. Zool.-Rev. Can. Zool..

[b55] Schjorring S (2001). Ecologically determined natal philopatry within a colony of great cormorants. Behav. Ecol..

[b56] Schjorring S, Gregersen J, Bregnballe T (2000). Sex difference in criteria determining fidelity towards breeding sites in the great cormorant. J. Anim. Ecol..

[b57] Schmutz JK, Robertson RJ, Cooke F (1983). Colonial nesting of the Hudson-Bay eider duck. Can. J. Zool.-Rev. Can. Zool..

[b58] Shorey L, Piertney S, Stone J, Hoglund J (2000). Fine-scale genetic structuring on *Manacus manacus* leks. Nature.

[b59] Siegel S, Castellan NJ (1988). Nonparametric statistics for the behavioral sciences.

[b60] Skabo S, Vaillancourt RE, Potts BM (1998). Fine-scale genetic structure of *Eucalyptus globulus* ssp. *globulus* forest revealed by RAPDs. Aust. J. Bot..

[b61] Smouse PE, Peakall R (1999). Spatial autocorrelation analysis of individual multiallele and multilocus genetic structure. Heredity.

[b62] Sonsthagen SA, Talbot SL, Lanctot RB, McCracken KG (2010). Do common eiders nest in kin groups? Microgeographic genetic structure in a philopatric sea duck. Mol. Ecol..

[b63] Stoen OG, Bellemain E, Saebo S, Swenson JE (2005). Kin-related spatial structure in brown bears *Ursus arctos*. Behav. Ecol. Sociobiol..

[b64] Taylor AC, Horsup A, Johnson CN, Sunnucks P, Sherwin B (1997). Relatedness structure detected by microsatellite analysis and attempted pedigree reconstruction in an endangered marsupial, the northern hairy-nosed wombat *Lasiorhinus krefftii*. Mol. Ecol..

[b65] Titman RD (1999). Red-breasted Merganser (*Mergus serrator*). The Birds of North America Online.

[b66] Uesugi R, Kunimoto Y, Osakabe M (2009). The fine-scale genetic structure of the two-spotted spider mite in a commercial greenhouse. Exp. Appl. Acarol..

[b67] Van der Jeugd HP, van der Veen IT, Larsson K (2002). Kin clustering in barnacle geese: familiarity or phenotype matching?. Behav. Ecol..

[b68] Waldeck P, Andersson M, Kilpi M, Öst M (2008). Spatial relatedness and brood parasitism in a female-philopatric bird population. Behav. Ecol..

[b69] Waldman B (1988). The ecology of kin recognition. Annu. Rev. Ecol. Syst..

[b70] Weller MW (1957). An automatic nest trap for waterfowl. J. Wildl. Manage..

[b71] Westerskov K (1950). Methods for determining the age of game bird eggs. J. Wildl. Manage..

[b72] Wojcieszek JM, Nicholls JA, Goldizen AW (2007). Stealing behavior and the maintenance of a visual display in the satin bowerbird. Behav. Ecol..

[b73] Wright S (1943). Isolation by distance. Genetics.

[b74] Yom-Tov Y (1974). Effect of food and predation on breeding density and success, clutch size and laying date of crow (Corvus-Corone L). J. Anim. Ecol..

[b75] Young AD, Titman RD (1988). Intraspecific nest parasitism in red-breasted mergansers. Can. J. Zool.-Rev. Can. Zool..

